# Cancer-targeted pro-theranostic bi-metallic organo-coordination nanoparticles

**DOI:** 10.7150/thno.99863

**Published:** 2025-01-01

**Authors:** Hengbo Huang, Lei Fang, Janaka Wansapura, Julie L. Prior, Brad Manion, Baogang Xu, Cody Hongsermeier, Nisha Gamadia, Nicole Blasi, Rui Tang, Christopher Egbulefu, Monica Shokeen, James D. Quirk, Samuel Achilefu

**Affiliations:** 1 Departments of Radiology, Washington University in St. Louis, MO 63110, USA.; 2 Departments of Biomedical Engineering, Washington University in St. Louis, MO 63110, USA.; 3 Advanced Imaging Research Center, University of Texas Southwestern Medical Center, Dallas, TX 75390, USA.; 4 Department of Biomedical Engineering, University of Texas Southwestern Medical Center, Dallas, TX 75235, USA.

**Keywords:** pro-theranostics, tannic acid, magnetic resonance imaging, near infrared fluorescence, ROS, radionuclide stimulated dynamic therapy

## Abstract

**Rationale:** Cancer remains a leading cause of mortality, with aggressive, treatment-resistant tumors posing significant challenges. Current combination therapies and imaging approaches often fail due to disparate pharmacokinetics and difficulties correlating drug delivery with therapeutic response.

**Methods:** In this study, we developed radionuclide-activatable theranostic nanoparticles (NPs) comprising folate receptor-targeted bimetallic organo-nanoparticles (Gd-Ti-FA-TA NPs). Polyvalent tannic acid was used to coordinate titanium (Ti), a reactive oxygen species (ROS)-generating catalyst, gadolinium (Gd), a magnetic resonance imaging (MRI) contrast agent, and cypate, a near-infrared fluorescent dye.

**Results:** The NPs exhibited higher magnetic field-dependent relaxivities (*r*_1_ = 20.8 mM⁻¹s⁻¹, *r*_2_ = 72.1 mM⁻¹s⁻¹) than Gd-DTPA (*r*_1_ = 4.8 mM⁻¹s⁻¹, *r*_2_ = 4.9 mM⁻¹s⁻¹) on a 3 T MRI scanner. Tannic acid coordination reduced the Ti band gap from 3.3 eV in TiO₂ NPs to 2.0 eV, tripling ROS generation under UV light exposure. In breast cancer models (4T1 and PyMT-Bo1), Cerenkov radiating radiopharmaceuticals activated Gd-Ti-FA-TA NPs *in vitro* and *in vivo*, generating cytotoxic ROS to inhibit tumor cell viability and prevent tumor progression. *In vivo*, the NPs selectively accumulated in 4T1 tumors and enhanced both T_1_ and T_2_ MRI contrast, highlighting a strategy to locally activate cytotoxic ROS generation with radiopharmaceuticals for cancer treatment, utilizing cross-modality PET/MRI and optical imaging for shallow and deep tissue visualization.

**Conclusion:** The integrated nanoplatform allows direct imaging of drug delivery, providing guidance for the optimal timeline to activate therapeutic effects of pro-theranostic NPs via external triggers such as radionuclide-stimulated dynamic treatment.

## Introduction

Despite advances in diagnosis and therapy, cancer is still one of the leading causes of death 1, 2. Promising therapies demonstrating initial remission have often resulted in cancer relapse, metastasis, and severe side effects. The inefficiency of single drugs to eradicate cancer cells has prompted the use of combination drugs, which have disparate biodistribution profiles in the body, creating uncertainty about whether these drugs reached their target. Imaging agents designed to image these events *in vivo* operate through mechanisms that are not necessarily aligned with those of the drugs. Furthermore, they are administered before or after therapy, effectively monitoring the drug effect but not its target efficacy. A potential strategy to overcome these impediments is the adoption of nanoparticles (NPs) for cancer diagnosis and treatment. The multifunctional capabilities of NPs offer unique opportunities to incorporate therapeutics and imaging agents for image-guided targeted therapies 3-6. Previous studies have demonstrated that these nanoplatforms can enhance the antitumor effect and reduce systemic side effects observed with standard therapies 7-10.

Metal-organic coordination networks are hybrid functional materials consisting of polyvalent organic metal chelators widely used in optics, electronics, energy, medicine, and biology 11-16. Their high porosity and large internal surface areas make them attractive for various applications, such as storage, sensors, catalysis, imaging, and drug delivery 17, 18. Tannic acid (TA), a naturally occurring polyphenol, has gained interest as an organic coordination component due to its abundant hydroxyl groups templated on a central glucose with five digalloyl moieties. The structural orientation of the branching galloyl moieties facilitates the formation of hydrogen bonds with proteins and small molecules or chelation with diverse metal ions 19.

Titanium dioxide (TiO_2_) is widely used in industry and medicine due to its light scattering and catalytic properties 20. Exposure to UV light can generate ROS for cell killing 21. To overcome the challenge of shallow UV light penetration in tissue, we previously demonstrated that Cerenkov-radiating radionuclides such as radiolabeled 2-fluorodeoxyglucose (^18^FDG) can interact with TiO_2_ NPs or other photosensitizers to generate cytotoxic ROS 22-24. We showed that this radionuclide-stimulated dynamic therapy (RaST) could inhibit tumor progression in animals and prevent metastasis 25. Chelating a radionuclide for positron emission tomography (PET), ^89^Zr, with TiO_2_ NPs allowed us to visualize the NPs' distribution *in vivo* and exert a therapeutic effect on tumors 26. However, attaching this radionuclide to TiO_2_ NPs spontaneously activated ROS, abrogating the beneficial spatiotemporal ROS activation in tumors during RaST. An alternative approach with magnetic resonance imaging (MRI) instead of PET could obviate this limitation.

MRI uses gadolinium (Gd) chelates as a contrast agent for T_1_-weight imaging due to its high paramagnetic properties 27, 28. However, the instability of Gd directly attached to TiO_2_ NPs released the toxic metal in solution, making it unfit for *in vivo* applications. TA, with its polyvalent digalloyl group and porosity, could stably chelate Gd to enhance MRI contrast while minimizing the toxic effects of free Gd 29, 30.

In this study, we used TA to develop pro-theranostic NPs. Pro-theranostics are stimuli-responsive, non-toxic materials or molecules that can provide imaging signals while remaining harmless under normal conditions. However, they possess the unique ability to transition into a toxic state when exposed to specific external or internal stimuli, enabling precise and localized therapeutic effects. The cancer-targeting pro-theranostic NPs used in this study incorporate Gd, Ti, and near-infrared (NIR) fluorescent dye into TA to form stable 200 nm NPs. NIR optical imaging and MRI allowed shallow and deep tissue imaging. We found that coordinating Ti with TA lowered the band gap to increase ROS generation and the induction of cell death. The high *r*_1_ and *r*_2_ relaxivities enhanced the potential applications of Gd-Ti-FA-TA NPs in various T_1_- and T_2_-weighted MRI. Embedding imaging agents into the pro-therapeutic NPs offers spatiotemporal image guidance to activate therapy at the optimal time point via externally triggered methods such as RaST.

## Material and Methods

### Chemicals

Gadolinium (III) chloride hexahydrate (GdCl_3_∙6H_2_O), titanium(IV) bis(ammonium lactato) dihydroxide solution (50 wt. % in H_2_O), poly(vinyl pyrrolidone) (PVP), tannic acid (TA), folic acid (FA, ≥97%), titanium (IV) oxide (TiO_2_ anatase, nanopowder, 25 nm particle size), formaldehyde solution (37 wt. % in H_2_O), and ethyl alcohol (anhydrous) were purchased from Sigma-Aldrich Co., St. Louis, MO, USA. Ammonium hydroxide (NH_4_OH, Certified ACS Plus) was purchased from Fisher Scientific International, Inc., Hampton, NH, USA. All chemicals were used as received. High-purity Milli-Q water (>18.2 MΩ·cm at 25 °C) was obtained from a Millipore water purification system (Millipore, Billerica, MA, USA). All solutions were freshly prepared for immediate use in each experiment.

### Preparation of Gd-Ti-FA-TA NPs

The Gd-Ti-FA-TA NPs were prepared by coordinating metal ions and TA following a modified literature protocol 16. Briefly, a typical synthesis consists of dissolving 33 mg PVP, 22 mg TA, and 0.3 mg FA in Milli-Q water (4.1 mL), followed by adding 0.9 mL EtOH. The solution was stirred at 300 rpm for 2 min. Ammonium (55 μL) and formaldehyde (42 μL) solutions were added to the mixture, and the solution was stirred at 300 rpm for 6 h. GdCl_3_ (55 mg/mL) was dissolved in Milli-Q water to prepare the working stock solution. To incorporate Gd and Ti onto TA, 200 µL GdCl_3_ stock solution and 40 μL titanium bis(ammonium lactato) dihydroxide solution (50 wt. % in H_2_O) were added to the TA solution. The mixture was stirred at room temperature for 12 h at 300 rpm, followed by incubation at 90 °C for 12 h with constant stirring. After the reaction, the Gd-Ti-TA NPs were washed with Milli-Q water by centrifugation (10,000 rpm, 10 min). The NPs were stored at 4 °C.

The Gd-FA-TA NPs and TiO_2_-Gd-FA-TA NPs were prepared following the same method used for Gd-Ti-TA NPs, except that we added (1) 200 µL GdCl_3_ (55 mg/mL) of the stock solution to the TA solution for Gd-FA-TA NPs synthesis, and (2) 200 µL GdCl_3_ stock solution (55 mg/mL) and 10 mg TiO_2_ anatase nanopowder (25 nm particle size) were added to the TA solution for TiO_2_-Gd-FA-TA NPs synthesis.

### Characterization of Gd-Ti-FA-TA NPs

Transmission electron microscopy (TEM) images of Gd-Ti-FA-TA NPs were captured using a JEM-1400 series 120kV Transmission Electron Microscope. Dynamic light scattering (DLS) measurements were acquired with a Malvern Zetasizer Nano ZS (Malvern Instruments Ltd., Malvern, UK) instrument equipped with a 633 nm laser. Three measurements were conducted for each sample with at least 10 runs, and each run lasted 10 s. All sizes reported were based on intensity average. The Gd and Ti elements on the purified NPs were quantified via a Perkin Elmer NexION 2000 Inductively Couple Mass Spectrometer (ICP-MS, Perkin Elmer, Inc., Waltham, MA, USA). The UV-vis spectra were acquired on a DU640 spectrophotometer (Beckman Coulter Inc, Brea, CA). The fluorescent spectra were acquired on a Horiba Fluorolog-3 Spectrofluorometer (HORIBA Instruments, Piscataway, NJ, USA). X-ray diffraction (XRD) data were obtained using a Rigaku microMax-003 X-ray generator equipped with a HyPix-6000 direct detector (Rigaku Americas Corporation, Woodlands, TX). X-ray photoelectron spectroscopy (XPS) measurements were performed with a VersaProbe II scanning X-ray photoelectron spectroscopy microprobe (Physical Electronics, Chanhassen, MN).

### Preparation of cypate-loaded Gd-Ti-FA-TA NPs

For NIR fluorescence imaging, cypate (1 μL, 20 mM, in DMSO) solution was added to 1 ml of the synthesized Gd-Ti-FA-TA NPs solution in Milli-Q water. The solution was then shaken for 30 min, followed by bath sonication for 15 min. The unbound cypate dye remained in the supernatant and was removed by centrifugation. The amount of cypate loaded was quantitatively evaluated by UV-VIS spectrophotometer (Beckman Coulter Inc, Brea, CA) with a molar extinction coefficient of 137,500 L mol^-1^ cm^-1^ at 779 nm.

### Measurement of band gap and ROS

Bandgap was determined using diffuse powder reflectance UV-vis spectroscopy (Horiba Fluorimeter and attached Quanta-ϕ integrating sphere, Kyoto, Japan). A Spectralon scattering blank (Labsphere Inc., North Sutton, NH) was used to calibrate the sphere before measuring dry-powdered Gd-Ti-TA NPs in a sample cup with a quartz coverslip and integration time of 0.5 sec.

ROS production was measured using 2'-7'-Dichlorofluorescein diacetate (DCF-DA) cell-free assay protocol 31. DCF-DA (90 μL, 5.55 mM) in DMSO was mixed with NaOH (10 μL, 1 M) to achieve a final concentration of 5 μM. The mixture was vortexed and placed in the dark for 15 min at room temperature for future use. DCF-DA mixture (8 μL) was then added to 1 mL of NPs (0.01 mg/ml Ti content, as determined by ICP-MS). About 150 μL of the solution was added into 3 wells of a black transparent bottom 96 well plate. The wells were then irradiated by a 365 nm UV lamp with an average UV power of 1 mW/cm^2^ during the activation stage. Samples were excited at 495 nm, and emission was detected at 525 nm (20 nm slit window) every 80 seconds for 30 min with Synergy Neo2 multi-mode reader (BioTek, Winooski, VT). Results were reported as pseudo-first order rate constants.

The stability of Gd-Ti-FA-TA NPs under UV light was determined by absorption spectra. Gd-Ti-FA-TA NPs were irradiated by a 365 nm UV lamp with an average UV power of 1 mW/cm^2^ for a time range from 5 min to 60 min. The spectra were acquired using the UV-VIS spectrophotometer (Beckman Coulter Inc, Brea, CA).

For RaST-mediated ROS production, ^18^FDG (150 μCi) was added to each solution containing activated DCF-DA and NPs in 96 well plates. The ROS produced was determined every minute for 20 min using Synergy LX multi-mode Reader (BioTek, Winooski, VT) equipped with a Green Filter (Ex 485/20 nm, Em 528/20 nm). The results were reported as pseudo first-order rate constants.

### Determination of cell viability

*In vitro* experiments were performed on 4T1 cells and PyMT-Bo1 cells as reported previously 32. Cells were cultured in Dulbecco's Modified Eagle's Medium containing 10% fetal bovine serum (FBS), L-glutamine (2 mM), penicillin (100 units/mL), and streptomycin (100 μg/mL), incubated at 37 °C in a humidified atmosphere of 5% CO_2_ and 95% air. Gd-Ti-FA-TA NPs were prepared as described above and redispersed in PBS to obtain a stock solution of 5 mg/mL. Cells were plated in 96-well plates with 8,000 cells per well and incubated for 24 h before adding 15.6-1,000 μg/mL Gd-Ti-FA-TA NPs to determine the concentration-dose response. ATP (adenosine triphosphate) cell viability assay, which determines the number of viable cells in culture by quantifying ATP, was performed using Cell Titer-Glo Cell Viability Assay (Promega Co., Fitchburg, WI, USA) according to the manufacturer's instructions. Briefly, ATP assay solution was added to each well and incubated at room temperature for 20 minutes before the relative luminescence read-out with the Synergy Neo2 multi-mode reader (BioTek, Winooski, VT). Incubation of Gd-Ti-FA-TA NPs in culture was spanned 1-3 days before analysis. Data was processed with the GraphPad Prism software.

MTT (3-(4, 5-dimethylthiazolyl-2)-2, 5-diphenyltetrazolium bromide) assay, a colorimetric assay measuring cellular mitochondrial activity, was performed in a similar manor. Briefly, after washing each well with 100 μL PBS to minimize the absorbance of NPs, 10 μL of 5 mg/mL thiazolyl blue tetrazolium bromide solution (Sigma-Aldrich Co., St. Louis, MO, USA) was added to each well and incubated at 37 °C for 4 h. The formazan crystals were then dissolved using 100 μL of solubilization solution (10 mL isopropanol + 420 μL 1 N HCl) before the absorbance (562 nm) read-out with the Synergy Neo2 multi-mode reader (BioTek, Winooski, VT). Incubation of Gd-Ti-FA-TA NPs in culture was spanned 1-3 days before analysis. Data was processed with the GraphPad Prism software.

### Internalization of NPs in cells

We followed published methods to culture these cells 33. Briefly, 4T1 and PyMT-Bo1 cells were seeded at 6000 cells per well in Lab-Tek 8-chambered slides (Nunc) and incubated for 24 h in a 37ºC CO_2_ incubator. Next, the cypate-loaded Gd-Ti-FA-TA NPs were added into the NP wells at 100 µg/mL concentration for 1, 4, and 24 h. After the treatment, the cells were washed three times with 1X PBS before staining with SYTO-13 nuclear stain (Invitrogen/molecular probes, Carlsbad, CA) for 45 min at 37 °C. Cells were mounted and coverslipped for epifluorescence for microscopy with 460-500/510-560 nm and 750-800/818-873 nm Ex/Em wavelengths for SYTO-13 and cypate, respectively. ImageJ software (National Institutes of Health) was used for image processing.

### *In vivo* tumor model

Seven-week-old male and female C57BL/6 mice purchased from Charles River Laboratories were housed and maintained according to the Washington University School of Medicine Animal Studies Committee. Animal studies were performed in accordance with the humane care and use of research animals. We used 4T1 tumor-bearing C57BL/6 mice for *in vivo* experiments following our previous method 32. Briefly, 4T1 cells (300,000 per mouse) were injected subcutaneously and grown until they reached 10 mm diameter, as determined by caliper measurements.

### *In vivo* NIR fluorescence imaging

We used 4T1 tumor-bearing C57BL/6 mice (n = 3), which were inoculated as described above, to determine the biodistribution of Gd-Ti-FA-TA NPs. The mice were shaved using commercially available hair removal equipment and products and anesthetized with isoflurane (inhalation of 2% isoflurane vaporized in oxygen). Freshly prepared cypate-loaded Gd-Ti-FA-TA NPs in PBS (100 μL, 1 mg/mL) were tail vein-injected and imaged at different time intervals (1, 2, 4, and 24 h). Fluorescence imaging was performed using excitation and emission wavelengths of 785 nm and 820 nm, respectively, in a Pearl Trilogy Small Animal Imaging System (Li-Cor Biosciences Inc.). Also, some tissues, including tumors, were excised and fluorescence imaged at 24 h post-injection with the Pearl Trilogy Small Animal Imaging System (Li-Cor Biosciences Inc., Lincoln, NE).

### Measurement of Gd-Ti-FA-TA and associated NPs relaxivity at different magnetic fields

The NP relaxivity was first measured with a 1.4 Tesla Pulsar NMR Spectrometer (Oxford Instruments, Oxfordshire, UK). Gd concentrations in the NPs were determined by ICP-MS. For each Gd concentration (0, 0.0375, 0.075, 0.15. 0.3 mM), 200 μL of the Gd-Ti-FA-TA NPs, Gd-FA-TA NPs, TiO_2_-Gd-FA-TA NPs, and Gd-DTPA (used as a reference) in MilliQ water were loaded into NMR tubes. The *r*_1_ and *r*_2_ were measured using an inversion recovery and a Carr-Purcell-Meiboom-Gill sequence, respectively 34, 35.

The MRI contrast and relaxivities of the NPs and Gd-DTPA were evaluated on a variable field PET/MR preclinical scanner operating at 3-Tesla (MR Solutions, Guildford, UK) using a 38 mm ID quadrature birdcage RF coil. MRI phantoms were made by loading different concentrations (0, 0.0375, 0.075, 0.15. 0.3 mM of Gd) of each NPs and Gd-DTPA into an 8-well plastic plate. For all sequences, a single 3 mm thick slice was acquired with a 6.4 × 3.2 cm field of view. MR images of the phantoms were acquired as follows. T_1_-weighted gradient echo: 256 × 128 matrix, 0.25 × 0.25 mm^2^ resolution, TR/TE = 100/3 ms, flip angle = 60°, 8 averages. T_2_-weighted fast spin echo images: 128 × 128 matrix, 0.25 × 0.5 mm^2^ resolution, TR/TE = 4000/15 ms, effective echo time = 75 ms, echo train length = 5, 4 averages. Fast spin echo inversion recovery images for T_1_ mapping: 128 × 64 matrix, 0.5 × 0.5 mm^2^ resolution, TR/TE = 8000/8 ms; echo train length = 4, TI = 10, 45, 200, 873, 4000 ms. Multi-echo spin echo for T_2_ mapping: 64 × 64 matrix, 0.5 × 1 mm^2^ resolution, TR/TE = 6000/15 ms; 16 echoes. *r*_1_ and *r*_2_ maps were generated for each imaging voxel by modeling as a mono-exponential decay using the Bayesian Analysis Toolbox (bayesiananalysis.wustl.edu) 36. In addition to 3 T, we measured the MRI parameters using these phantoms on a 4.7 T Direct preclinical scanner (Agilent Technologies, Santa Clara, CA, USA) and on the variable field PET/MR operating at 7 T.

### *In vivo* magnetic resonance imaging

4T1 tumor-bearing C57BL/6 mice were used for *in vivo* intratumoral and intravenous injections of the NPs and MRI. To increase the persistence of intratumorally injected Gd-Ti-FA-TA NPs in tumors, a sol-gel solution was prepared by dispersing the NPs in 20 wt% Pluronic F127 (Sigma Aldrich Co., St. Louis, MO, USA) to form a gel structure with a final concentration of 100 μg/mL. About 40 μL of the gel was injected into multiple regions in the tumor (n = 3). MRI scans were carried out immediately after the injection using the variable field PET/MR preclinical scanner measure (MR Solutions, Guildford, UK) operating at 3-Tesla. T_1_-weighted gradient echo images: 128 × 128 matrix; 16 slices with 1 mm each; 0.25 × 0.25 mm^2^ resolution, TR/TE = 1000/3 ms, 8 averages, flip angle = 30°. T_2_-weighted fast spin echo images: 256 × 128 matrix; 16 slices with 1 mm each; 0.127 x 0.25 mm^2^ resolution, TR/TE = 2000/13 ms, effective echo time = 52 ms, echo train length = 4; 1 average.

For systemic administration, Gd-Ti-FA-TA NP (100 μL, 1 mg/mL in PBS, n = 3) was injected intravenously via the tail vein, as described in the fluorescence imaging section. The MRI scan of the mice was performed under anesthesia on a 3 T Bruker Biospec Preclinical MR scanner (Bruker, Billerica, MA). Fast low angle shot (FLASH) gradient echo for T_1_-weighted images: 128 × 128 matrix; 32 slices with 1 mm each; 0.25 × 0.25 mm^2^ resolution, TR/TE = 400/4 ms, 5 averages, flip angle = 30°. Data were analyzed in ParaVision 360 and ImageJ software.

### *In vivo* RaST in 4T1 tumor-bearing mice

Six-week-old C57BL/6 mice were injected subcutaneously on the right flank with 5×10^5^ 4T1-GFP-luc cells (n=25 mice). Using BLI signal to determine tumor engraftment and viability, the mice were assigned into 4 groups (n = 8, 7, 5, 5), on day 5 after the tumor implantation. Mice in each group received intravenous injection of (1) Gd-Ti-FA-TA NPs (100 μL of 1 mg/ml) and ^18^FDG (600 μCi), (n = 8); (2) Gd-Ti-FA-TA NPs alone (100 μ of 1 mg/ml, n = 7); (3) ^18^FDG alone (600 μCi, 100 μ of 1 mg/ml, n = 5); and (4) untreated (n = 5). Treatments were given twice a week over a 15-day period starting from day 5 post-tumor implantation, where a treatment refers to an administration of Gd-Ti-FA-TA NPs and/or ^18^FDG. Typically, for a treatment with both NPs and^ 18^FDG, mice were administered with 100 μL of Gd-Ti-FA-TA NPs (1 mg/ml) intravenously, followed by 600 μCi^ 18^FDG 18 hours later, which was also administrated intravenously. BLI, body weight, and caliper measurements were performed for all the mice on the same day as the administration of Gd-Ti-FA-TA NPs. BLI was used to determine the level of viable cells, and caliper measurements were used to determine the tumor volume was determined with calipers using the formula 1/2 × length × (width)^2^. On day 16, all mice were euthanized, and their tumors were collected and weighed.

## Results and Discussion

### Tannic acid stabilizes titanium and gadolinium metals in spherical nanoparticles

**Figure 1:**
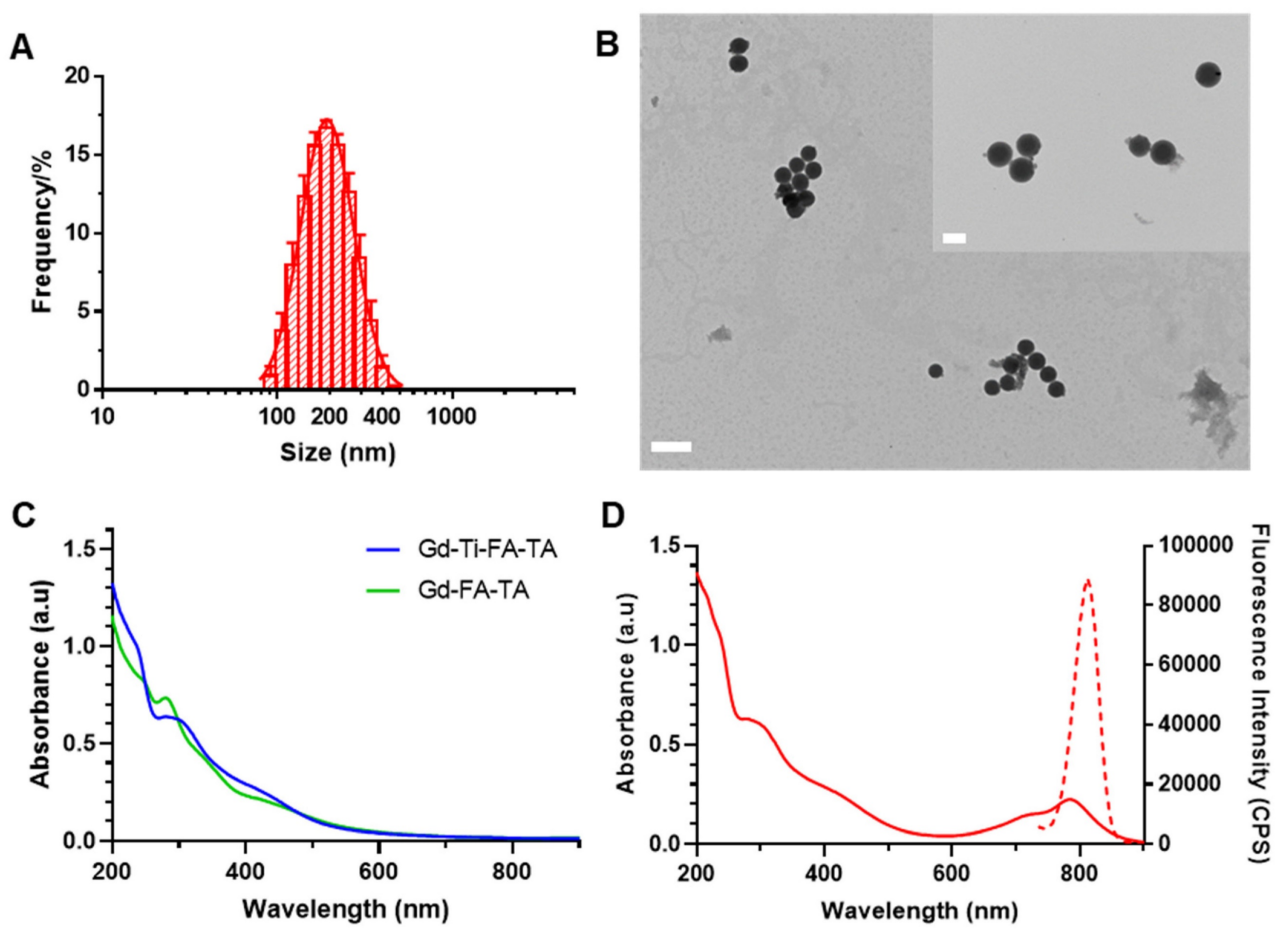
** Characterization of Gd-Ti-FA-TA NPs.** (A) Gd-Ti-FA-TA NPs particle size determination by DLS. (B) Representative TEM images of Gd-Ti-FA-TA NPs. (Scale bar: 500 nm, inner scale bar: 200 nm.) (C) UV-vis absorption spectra of Gd-Ti-FA-TA NPs and Gd-FA-TA NPs. (D) UV-vis absorption and fluorescence spectra of cypate-loaded Gd-Ti-FA-TA NPs.

The multiple polyphenolic groups of tannic acid enable the binding of diverse metals and the adsorption of organic molecules to form stable NPs 37. Leveraging this functionality, we functionalized TA with folic acid to target folate receptors on extracellular membranes of cancer cells 38. Coordination of Gd and Ti with the folate-targeting TA formed Gd-Ti-FA-TA NPs. Separately, Gd-FA-TA NPs, TiO_2_ NPs, and TiO_2_-Gd-FA-TA NPs were also prepared to serve as a reference in this study. We also adsorbed cypate dye onto the NPs to facilitate cellular and tissue imaging. Cypate is an NIR fluorescent dye widely used in biomedical imaging applications due to its excellent optical properties 39. Combined with Gd, the multifunctional NPs are capable of imaging cells and shallow tissues with high spatial resolution and quantitative deep tissue imaging. DLS analysis showed Gd-Ti-FA-TA NPs size of 224.7 ± 5.8 nm by intensity (Fig. 1A), with a polydispersity index (PDI) of 0.152 ± 0.008, indicating a good level of monodispersity. TEM revealed spherical structures with an average size of 211.1 ± 14.3 nm (Fig. 1B), a value that is slightly smaller than DLS due to the absence of solvation effect. The DLS and TEM images were also acquired for Gd-FA-TA NPs, TiO_2_ NPs, and TiO_2_-Gd-FA-TA NPs (Fig. S1). The UV-vis spectra peaks for TA at 214 nm and 276 nm (Fig. S2) were slightly red-shifted to 235 nm and 295 nm and broadened in the Gd-Ti-FA-TA NPS, indicating coordination between the metals and TA (Fig. 1C). In addition, the broad peak observed around 425 nm in the Gd-Ti-FA-TA NPs spectra corresponds to ligand-to-metal charge transfer, further confirming the formation of coordination complexes between the metal ions and TA. Cypate's fluorescence in Gd-Ti-FA-TA NPs displayed a 10 nm bathochromic shift (Fig. 1D) compared to the free dye (from 800 to 810 nm). While this shift could be attributable to several factors, such as enhanced π-π conjugation or extended hydrogen bonding, the significant broadening of cypate's absorption spectrum suggests the NP environment promoted the dye aggregation. Sequestration of these aggregates inside the hydrophobic phenylic interior further stabilizes the excited state of cypate, potentially contributing to the observed bathochromic shift. Consistency in the absorption spectra after UV light irradiation of the Gd-Ti-FA-TA NPs for up to 60 min demonstrated their high stability under these conditions (Fig. S3). X-ray diffraction (XRD) showed that Gd-Ti-FA-TA NPs retained an amorphous structure (Fig. S4). ICP-MS confirmed the composition of Gd and Ti. For a typical synthesis, the Gd-Ti-FA-TA NPs comprise Gd (0.68 ± 0.05 mg/mL; 4.32 mM) and Ti (0.62 ± 0.05 mg/mL; 12.95 mM), giving a molar ratio of about 1:3 for these metals in TA. Given the role of Ti as a photosensitizer in this construct, its higher molar concentration than Gd is desirable for therapy induction. The presence of Gd and Ti in the Gd-Ti-FA-TA NPs was further confirmed by XPS (Fig. S5). The Ti 2p spectrum exhibited two peaks at 464.6 eV and 458.8 eV, indicating that Ti predominantly existed in the +4 oxidation state within the NPs 40. The Gd 4d spectrum showed peaks at 148.8 eV and 143.2 eV, while the Gd 3d spectrum displayed peaks at 1188.8 eV and 1222.8 eV, corresponding to the 3d_5/2_ and 3d_3/2_ states, respectively. These peaks were attributed to Gd³⁺-O binding, further supporting the formation of coordination bonds between Gd and the ligands 41.

### Folate receptor mediates internalization of Gd-Ti-FA-TA NPs in breast cancer cells

**Figure 2:**
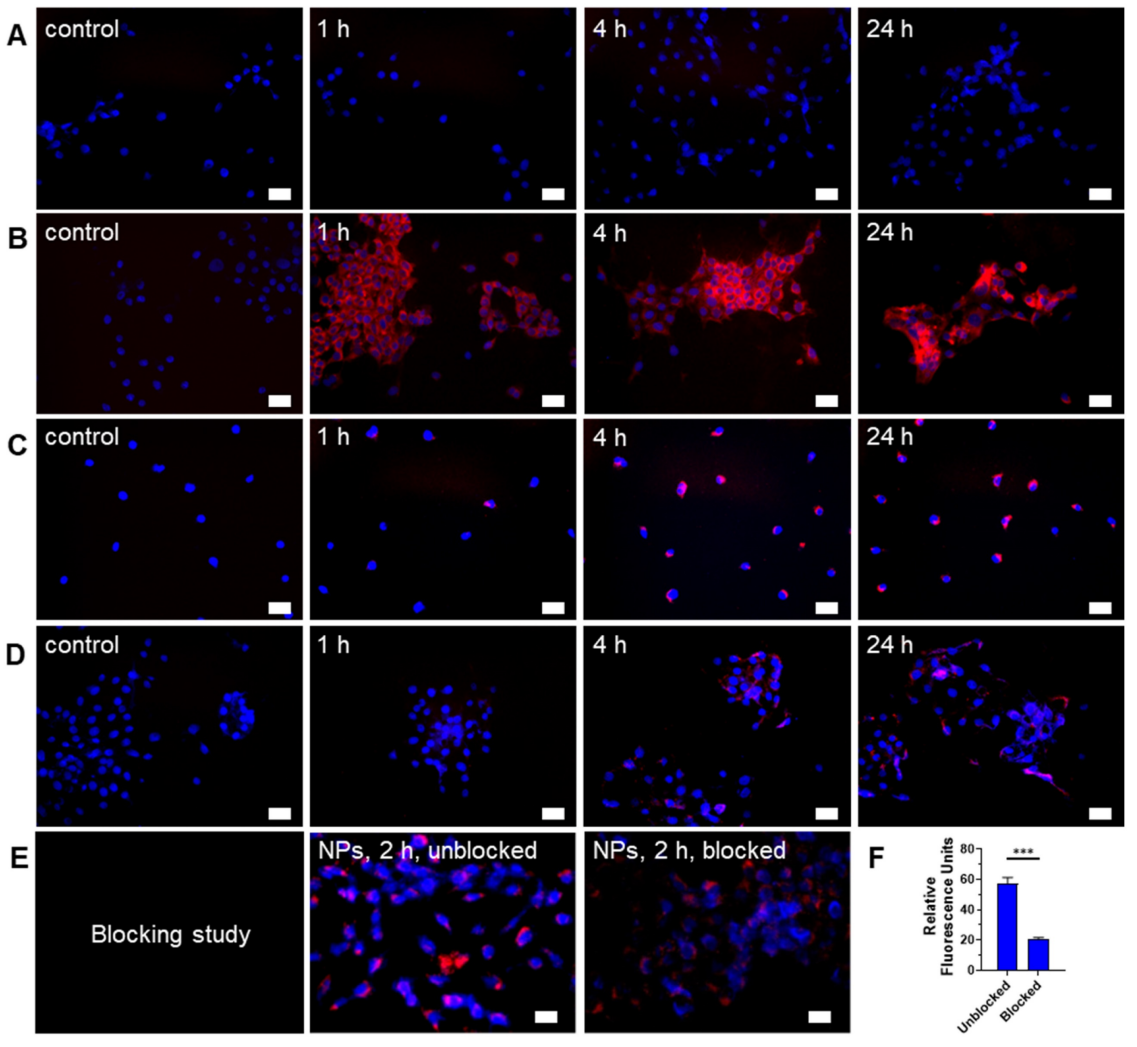
** Fluorescence microscopy of Gd-Ti-FA-TA NPs, Gd-Ti-TA NPs, and cypate uptake in breast cancer cell**s. (A) Cell internalization of 5 μM cypate by 4T1 cells after incubating for 1, 4, and 24 h. (B) 4T1 cell and (C) PyMT-Bo1 cell internalization of 30 μg/mL cypate-loaded Gd-Ti-FA-TA after incubating for 1, 4, and 24 h. (D) Cell internalization of non-folic acid-conjugated cypate-loaded Gd-Ti-TA NPs by 4T1 cells after incubating for 1, 4, and 24 h. (E) Blocking of Gd-Ti-FA-TA NP internalization by 4T1 cells after first incubating with 200 μM folic acid, followed by adding and incubating with Gd-Ti-FA-TA NPs for 2 h. (F) Quantitative fluorescence analysis demonstrates inhibition of NPs internalization, consistent with blocking folate receptors. Blue: DAPI, red: NIR fluorescence (cypate signal).

Depending on design considerations, NPs can internalize in cells by passive or active transport mechanisms. Passive NPs accumulation in tumors takes advantage of the enhanced permeability and retention (EPR) effect mediated by the leaky tumor vasculature and impaired lymphatic drainage 42. To facilitate active transport, we incorporated folic acid, which targets folate receptors, into the NPs. Most cancer cells and solid tumors, including 4T1 and PyMT-Bo1 cells, overexpress this receptor, making it a good candidate for folic acid-driven targeted drug delivery 43. The NIR fluorescent dye, cypate 32, facilitated the imaging of Gd-Ti-FA-TA NPs internalization in these cells. While cypate dye alone (5 μM) was not retained in 4T1 cells after 24-h incubation (Fig. 2A), the cancer-targeted NPs loaded with the same concentration of cypate exhibited a cell-type dependent uptake. 4T1 cells rapidly internalized the NPs within 1 h, which increased over time and displayed diffuse cytosolic fluorescence, but the uptake kinetics in PyMT-Bo1 cells were slow and focal (Fig. 2B,C). This cellular distribution pattern suggests that 4T1 internalized the NPs by both active and passive mechanisms. To estimate the contributions of both pathways, we incubated the non-folic acid-conjugated cypate-loaded Gd-Ti-TA NPs with 4T1 cells. Compared to the folate-conjugated NPs, these Gd-Ti-TA NPs internalized slowly, becoming detectable after 4-h incubation, attaining only 18% of the folate-targeted Gd-Ti-FA-TA NPs after 24-h incubation at the same microscope settings (Fig. 2D). Furthermore, pre-treating 4T1 cells with 200 μM of folic acid for 18 h at 37 °C to block folate receptors on cancer cells, followed by incubating with Gd-Ti-FA-TA NPs showed effective inhibition of NPs internalization (Fig. 2E), achieving about 80% blocking (Fig. 2F). These results demonstrate that 4T1 predominantly internalized Gd-Ti-FA-TA NPs via folate receptor-mediated endocytosis. Gd-Ti-TA NPs and the blocking studies suggest that about 20% of uptake could be attributed to a passive internalization pathway.

### UV light and RaST stimulate Gd-Ti-FA-TA NPs ROS generation

ROS, which includes superoxide anion (O_2_^•-^), hydrogen peroxide (HO), and hydroxyl radicals (⋅OH), play a critical role in normal human physiology but become cytotoxic at high levels. Organic and inorganic photosensitizers such as porphyrins 44 and TiO_2_ NPs are used to potentiate ROS generation in tumors, leading to the oxidative damage of lipids, proteins, DNA, and other cellular components 45, 46. A variety of techniques have been used to stimulate ROS generation in cells and tissues, including light, X-ray, ultrasound, and Cerenkov-emitting radionuclides (RaST), with the last three overcoming the tissue-depth limitation of light 22. In this study, we postulated that incorporating Ti into TA would enhance ROS generation.

**Figure 3:**
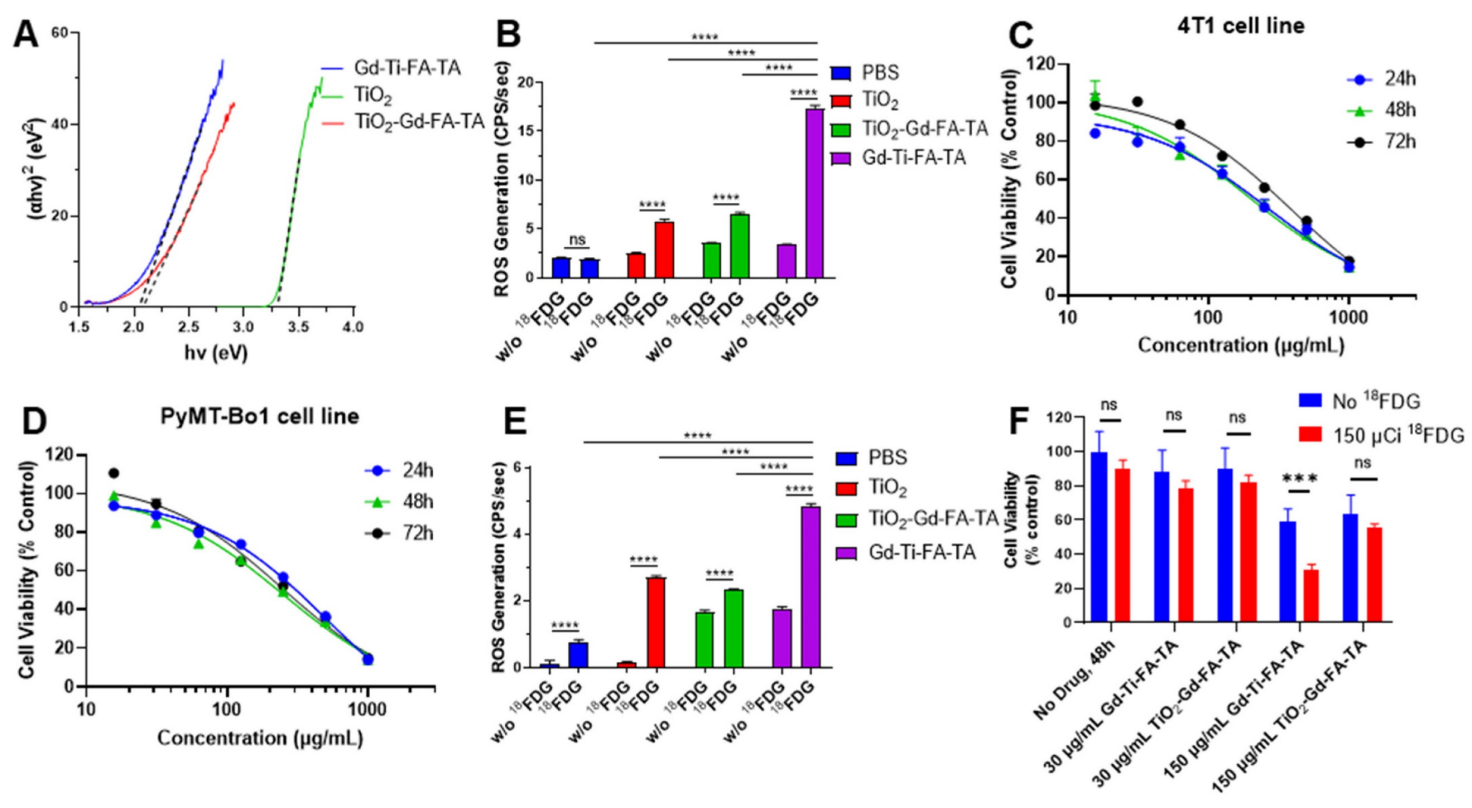
** Band gap, stimulated ROS generation, and cytotoxicity of NPs.** (A) Tauc plots to determine band gaps of Gd-Ti-FA-TA NPs, TiO_2_-Gd-FA-TA NPs, and anatase TiO_2_ NPs. (B) ROS generation by Gd-Ti-FA-TA NPs, TiO_2_-Gd-FA-TA NPs, anatase TiO_2_ NPs, and PBS with and without UV light. (C) 4T1 and (D) PyMT-Bo1 cell viability after incubating with Gd-Ti-FA-TA NPs using ATP assay. (E) ROS generation by Gd-Ti-FA-TA NPs, TiO_2_-Gd-FA-TA NPs, anatase TiO_2_ NPs, and PBS with and without 150 μCi ^18^FDG. (F) 4T1 cell viability after incubating with Gd-Ti-FA-TA NPs and TiO_2_-Gd-FA-TA NPs (30 and 150 μg/mL). The cells were treated with and without ^18^FDG (150 μCi per well) to simulate RaST; *p* = 0.00034.

A direct relationship exists between band gap and ROS generation, as the former determines the photon absorption range for the materials, which is necessary to create electron-hole pairs for ROS production. Thus, we first determined the Gd-Ti-FA-TA NPs band gap with reference to TiO_2_ NPs and anatase TiO_2_-Gd-FA-TA NPs. While the measured band gap for anatase TiO_2,_ a regenerative ROS catalyst, was 3.30 eV, coordination of Ti and anatase TiO_2_ with TA significantly lowered the NPs band gap to 2.04 eV and 2.08 eV, respectively (Fig. 3A). By increasing the range of photons absorbed and the number of electron-hole pairs, more excited electrons can oxidize molecular oxygen to O₂⁻ (and subsequently ⋅OH) while the holes can oxidize water directly to the highly reactive ⋅OH. As predicted, the ROS generation rate of Gd-Ti-FA-TA NPs was 17.39 CPS/sec, which is 3 times higher than the measured 5.824 CPS/sec for anatase TiO_2_ NPs (Fig. 3B). Despite the reduced band gap for TiO_2_-Gd-FA-TA NPs, the ROS generation rate (6.58 CPS/sec) was only slightly higher than TiO_2_ NPs but significantly lower than Gd-Ti-FA-TA NPs. This discrepancy could be attributed to multiple factors, including differences in the surface area and accessible catalytic site, the electron-hole pair dynamics, and the mode of interaction with tannic acid 47, 48. Particularly, the anatase crystalline structure could constrain the number of accessible sites for ROS generation compared to Ti. The lower band gap and superior ROS generation capacity of Gd-Ti-FA-TA NPs highlight the prospect of using them as photosensitizers for RaST or other activation methods.

### Radionuclide potentiates the cytotoxic effects of Gd-Ti-FA-TA NPs

The pro-theranostic NPs are designed to trigger ROS production for cancer killing. By targeting and selectively activating these NPs in tumors, we expect them to spare healthy tissues while exerting therapeutic effects on cancer.

Hence, we used ATP assay, which measures intracellular ATP levels, to determine the half-maximal inhibitory concentration (IC_50_) of Gd-Ti-FA-TA in 4T1 and PyMT-Bo1 cells. The NPs concentrations employed ranged from 15.6-1,000 µg/mL of the NPs at various time points. At 24 h, the IC_50_ of Gd-Ti-FA-TA NPs in 4T1 cells was 263 µg/mL, increasing to 396 µg/mL at 72 h (Fig. 3C). The opposite trend was observed in the PyMT-Bo1 cell line, where the IC_50_ decreased from 498 µg/mL to 247 µg/mL at 24 and 72 h, respectively (Fig. 3D). These data suggest the adaptation of 4T1 cells to the NPs over time, leading to a more resistant phenotype. Conversely, PyMT-Bo1 cells were more susceptible to the NPs, enabling the killing of these cells at lower NP concentrations. Although both cell lines are known to be resistant to diverse treatment methods 49, 50, the triple-negative breast cancer cells, 4T1, is one of the well-studied chemotherapy and radiotherapy-resistant cells.

The increase in IC_50_ over time for 4T1 cells treated with Gd-Ti-FA-TA NPs observed in the ATP assay suggests a potential recovery of these cells following initial stress. To further evaluate the cytotoxicity of Gd-Ti-FA-TA NPs, we cross-validated the ATP assay with the MTT assay, which measures mitochondrial activity in living cells. The calculated IC_50_ values from the MTT assay for Gd-Ti-FA-TA NPs in 4T1 cells were 1003, 883, and 633 µg/mL at 24, 48, and 72 h, respectively (Fig. S6A). Similarly, the IC_50_ of Gd-Ti-FA-TA NPs of PyMT-Bo1 cells were 1179, 666, and 560 µg/mL at 24, 48 and 72 h, respectively (Fig. S6B). Unlike the ATP assay result, cell viability decreased with time in the MTT assay, but both assays demonstrated low cytotoxicity of the NPs, even at high concentrations. However, the contrasting time-dependent increase in IC_50_ observed in the ATP assay versus the opposite trend in the MTT assay highlights the complexity of cellular responses to NPs and reflects the distinct biological processes measured by these assays over time. As recently reported, NPs can mediate cell death through multiple pathways, including apoptosis, necroptosis, and necrosis 51. Early-stage apoptotic cells may show reduced mitochondrial activity (detected by the MTT assay) while maintaining ATP levels in these and unaffected viable cells (detected by the ATP assay). Despite varying levels of therapy resistance, the high IC_50_ values observed in both cell lines indicate that Gd-Ti-FA-TA NPs are not effective for therapeutic purposes without an additional stimulus.

We explored whether radiopharmaceuticals could serve as an external activator of cytotoxic ROS generation in cells during RaST 22. We first conducted a ROS generation study with radionuclide ^18^FDG. The ROS generation rate for Gd-Ti-FA-TA NPs was 4.819 CPS/sec, which is significantly higher than the 2.705 CPS/sec for anatase TiO_2_ NPs (Fig. 3E). Guided by the cytotoxicity data, we incubated 4T1 cells with two Gd-FA-TA NP concentrations below the IC_50_ (30 and 150 μg/mL), followed by the addition of ^18^FDG. In the absence of ^18^FDG, a statistically insignificant decrease in 4T1 cell viability was observed with the low 30 μg/mL NP treatment (Fig. 3F). The higher 150 μg/mL Gd-Ti-FA-TA NP concentration initiated mild cell death, which was potentiated by ^18^FDG to about 30% viable cells. Consistent with the low ROS-generating capacity TiO_2_-Gd-FA-TA NPs, ^18^FDG treatment at the same high NP concentration did not induce significant cell death.

These results demonstrate that the low energy band gap and accessibility of Ti in the tannic acid complex enhanced ROS production, which translated into efficient cancer cell death. RaST appears to potentiate therapeutic effects after the NPs initiated cell death. Future studies will determine the biological underpinning for this complementary cell death.

### Gd-Ti-FA-TA NPs selectively accumulated in tumors *in vivo*

**Figure 4:**
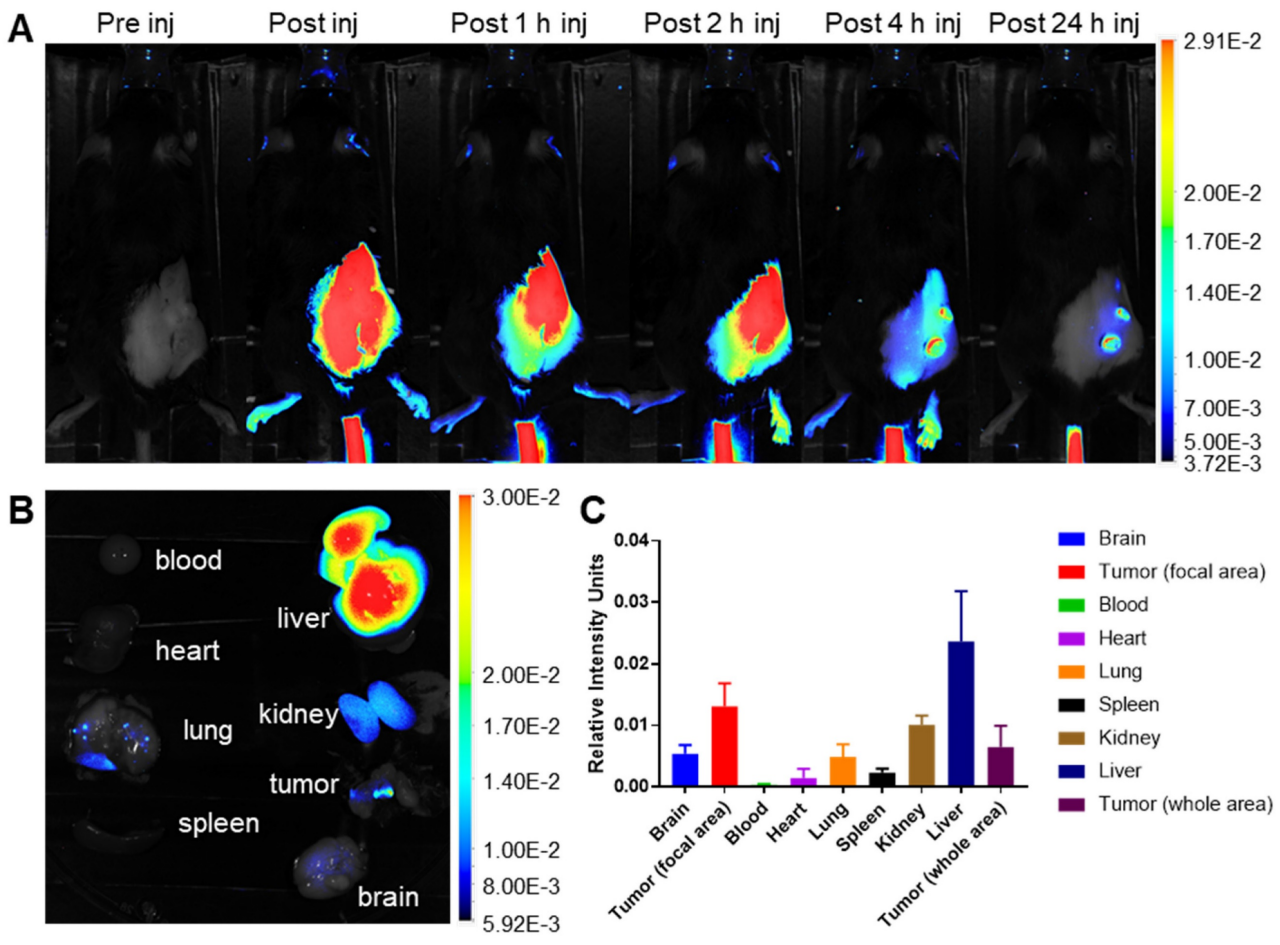
***In vivo* and *ex vivo* fluorescence imaging of cypate-loaded Gd-Ti-FA-TA NP distribution.** (A) Dorsal view fluorescence images of representative mouse after tail vein injection of cypate-loaded Gd-Ti-FA-TA NPs. Images were acquired pre-injection, immediately post-injection, and 1, 2, 4, and 24 h post-injection. (B) *Ex vivo* fluorescence biodistribution of major organs after mouse euthanasia 24 h post-injection of the NPs. (C) Relative fluorescence intensity of major organs *ex vivo* at 24 h post-injection of the NPs.

A significant challenge in cancer treatment is ensuring that drugs reach their intended targets. The integration of imaging and therapeutic agents in cypate-adsorbed Gd-Ti-FA-TA nanoparticles (NPs) allow for precise imaging to determine prodrug distribution. To evaluate the *in vivo* behavior of these NPs, we developed subcutaneous 4T1 tumors by injecting 300,000 cells subcutaneously in C57BL/6 mice. Tumor growth was monitored using calipers to initiate imaging when tumors reached 10 mm. After tail vein injection of Gd-Ti-FA-TA NPs (100 μL, 1 mg/mL planar, n = 3), planar fluorescence imaging revealed that the NPs rapidly distributed throughout the body but significantly cleared within 4 h post-injection (Fig. 4A and Fig. S7A). Due to fluorescence attenuation by animal fur, only shaved areas were visible. By 24 h post-injection, the nanoparticles were predominantly localized in focal areas of the tumor, liver, kidneys, and lungs (Fig. 4B,C). Tumor uptake was notably confined to two lateral regions, possibly due to high folate receptor density or EPR effect. Future studies could focus on elucidating these pathways *in vivo*.

### Gd-Ti-FA-TA NPs exhibited magnetic field-dependent *r*_1_ and *r*_2_ relaxivities for dual-modality MRI contrast

Gd is widely used as an MRI contrast agent due to its strong paramagnetic properties. Typically, Gd is administered as a metal chelate to prevent toxicity, which can affect its *r*_1_ and *r*_2_ relaxivities. In this study, we leveraged TA's multiple phenolic groups to stably chelate Gd without compromising the stability of Ti. MR scanners with different field strengths are used clinically, but the relationship between Gd relaxivities and the magnetic field strength is complex with multiple contributions, including exchange and correlation times. We determined the *r*_1_ and *r*_2_ relaxivities of Gd-Ti-FA-TA NPs, Gd-FA-TA NPs, TiO₂-Gd-FA-TA NPs, and commercial Gd-DTPA at 1.4 T (relaxometer), 3 T, 4.7 T, and 7 T. At 1.4 T, the *r*_1_ and *r*_2_ values for Gd-Ti-FA-TA (25.6 and 41.0 mM⁻¹s⁻¹), Gd-FA-TA (34.3 and 63.2 mM⁻¹s⁻¹), and TiO_2_-Gd-FA-TA (23.6 and 34.1 mM⁻¹s⁻¹) NPs were significantly higher than those of Gd-DTPA (3.6 and 4.3 mM⁻¹s⁻¹). Chelation with TA increased the relaxivities of Gd by more than threefold in all cases. This enhancement predicts a significant improvement in the MRI contrast properties of the NPs. The NPs exhibited a higher r2/r1 ratio than Gd-DTPA, indicating a greater enhancement in T_2_- than T_1_-weighted MR imaging. We observed that including Ti or TiO₂ in the NPs resulted in a noticeable decrease in both *r*_1_ and *r*_2_ relaxivities. This reduction is likely due to competitive binding and interactions between Ti and Gd ions with TA, possibly altering the spatial arrangement of Gd ions or introducing steric hindrance, which limits the accessibility of water molecules to the Gd ions.

**Figure 5:**
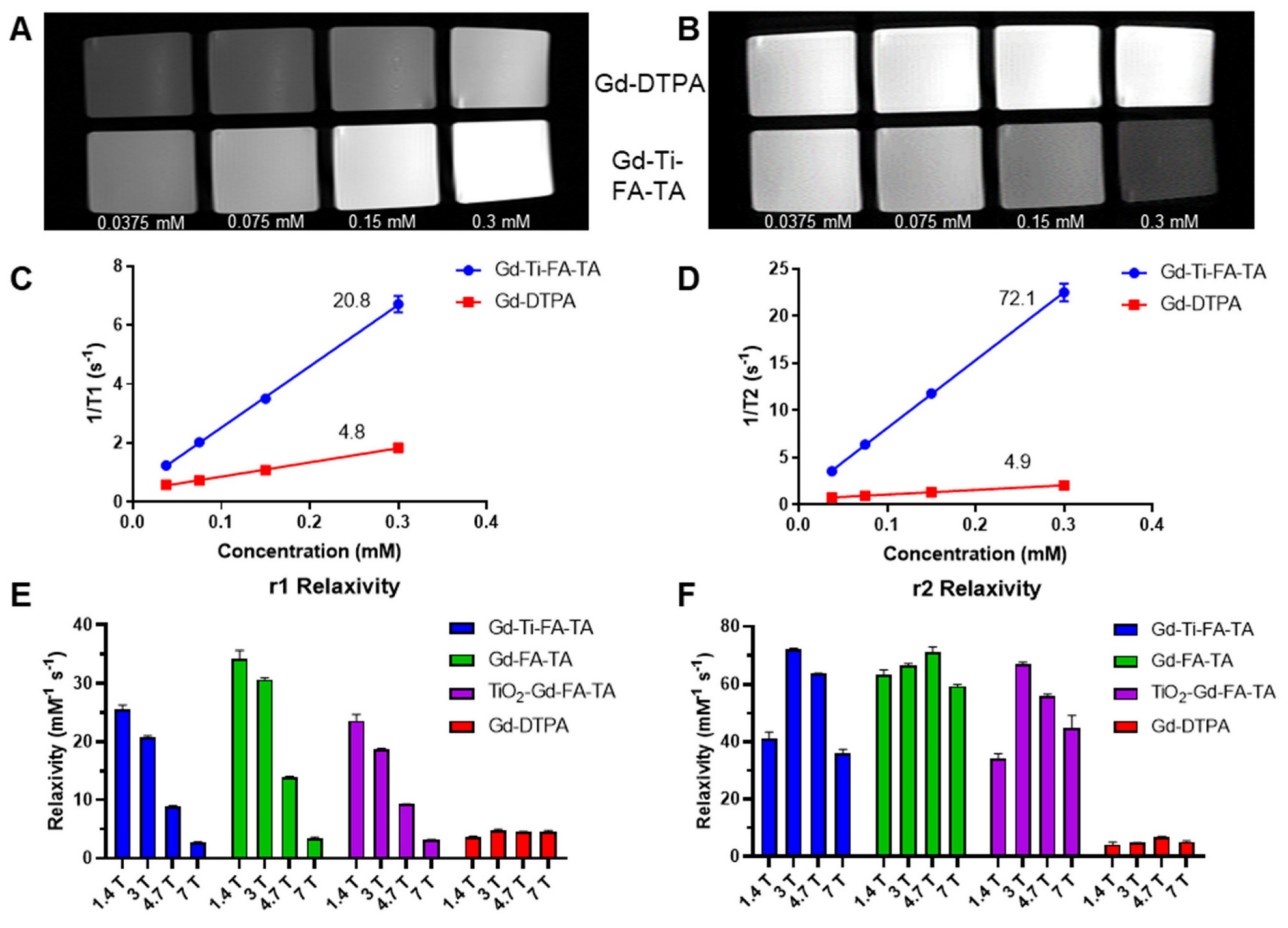
** MR contrast and parameters of TA-chelated Gd NPs at different field strengths.** (A) T_1_- and (B) T_2_-weighted images were acquired on a 3 T MR preclinical scanner with respective sequences. Gd-Ti-FA-TA NPs were plated with different concentrations (0.0375, 0.075, 0.15, and 0.3 mM) based on Gd concentration determined with ICP-MS. Gd-DTPA was used as a reference. (C) *r*_1_ relaxivity and (D) *r*_2_ relaxivity of Gd-Ti-FA-TA NPs were extracted from T_1_ and T_2_ mapping on a 3 T MR preclinical scanner. Gd-DTPA was used as a reference. (E) *r*_1_ relaxivity and (F) *r*_2_ relaxivity of Gd-Ti-FA-TA NPs at different magnetic fields. The same procedure was used for Gd-FA-TA and TiO_2_-Gd-FA-TA NPs.

Next, we developed an MRI phantom to assess T_1_ and T_2_ contrast enhancement and extract relaxivity parameters from a 3 T preclinical PET/MR scanner. We determined the Gd concentration using ICP-MS to determine the relaxivity per Gd ion. T_1_- and T_2_-weighted imaging sequences with Gd-Ti-FA-TA NPs and Gd-DTPA at different concentrations were performed (Fig. 5A,B). The Gd-Ti-FA-TA NPs significantly enhanced both T_1_ and T_2_ contrast, producing brighter T_1_-weighted and darker T_2_-weighted images than Gd-DTPA. Extracted *r*_1_ and *r*_2_ values from the T_1_ and T_2_ maps at 3 T were 20.8 and 72.1 mM⁻¹s⁻¹ for Gd-Ti-FA-TA NPs, compared to 4.8 and 4.9 mM⁻¹s⁻¹ for Gd-DTPA (Fig. 5C,D). These values were consistent with the trend observed at 1.4 T, except that *r*_1_ decreased by 20% while *r*_2_ increased by 76% at 3 T. The opposing effects at different field strengths align with the mechanisms of contrast generation. Lower fields optimize dipole-dipole interactions through maximization of rotational correlation and electronic relaxation time, leading to higher *r*_1_ values at 1.4 T. This trend continued as field strength increased, with *r*_1_ decreasing to 8.9 and 2.8 mM⁻¹s⁻¹ at 4.7 T and 7 T, respectively (Fig. 5E,F). The *r*_1_ at 7 T is smaller than that of Gd-DTPA, suggesting that Gd-Ti-FA-TA NPs are not suitable for T_1_-weighted MRI at this field strength. Conversely, the magnetic susceptibility differences between Gd ions and surrounding tissue at higher magnetic fields are more pronounced, leading to increased local field inhomogeneities and enhanced dephasing of water proton spins. This condition significantly increased *r*_2_ at 3 T compared to 1.5 T. However, the *r*_2_ of Gd-Ti-FA-TA NPs decreased to 63.8 and 36.1 mM⁻¹s⁻¹ at 4.7 T and 7 T, respectively, from a high of 72.1 mM⁻¹s⁻¹ at 3 T, highlighting the nuanced relationship between field strength and relaxivity.

Extending the *r*_1_ and *r*_2_ measurements to Gd-FA-TA and TiO_2_-Gd-FA-TA NPs revealed a similar trend as Gd-Ti-FA-TA (Fig. 5E,F). The Gd-Ti-FA-TA and TiO_2_-Gd-FA-TA values were more closely aligned than those of Gd-FA-TA. Perturbations in the local electronic configuration and shielding effects by Ti likely altered water exchange dynamics and water proton spin dephasing, contributing to the differential response observed. Despite these differences, the *r*_2_/*r*_1_ ratio for all TA-coordinated Gd complexes increased proportionally with magnetic field strength, whereas the reference Gd-DTPA values exhibited minimal changes (Table 1). Our results highlight the complex interplay between multiple metal ions within the nanoparticle structure and their impact on MRI contrast, revealing a dominant T_2_-weighted contrast enhancement for all Gd-TA NPs. Despite efforts to improve the *r*_1_ relaxivity of traditional T_2_ imaging agents such as iron oxide NPs 52-54, they are typically much lower than the values obtained with the Gd-Ti-FA-TA NPs developed in this study. For example, the high r1 relaxivity at 3 T confers dual T_1_ and T_2_ MRI capability on these Gd-FA-TA NPs.

### Gd-Ti-FA-TA NPs can serve as cancer-targeted dual modality T_1_ and T_2_ MRI contrast agents

**Figure 6:**
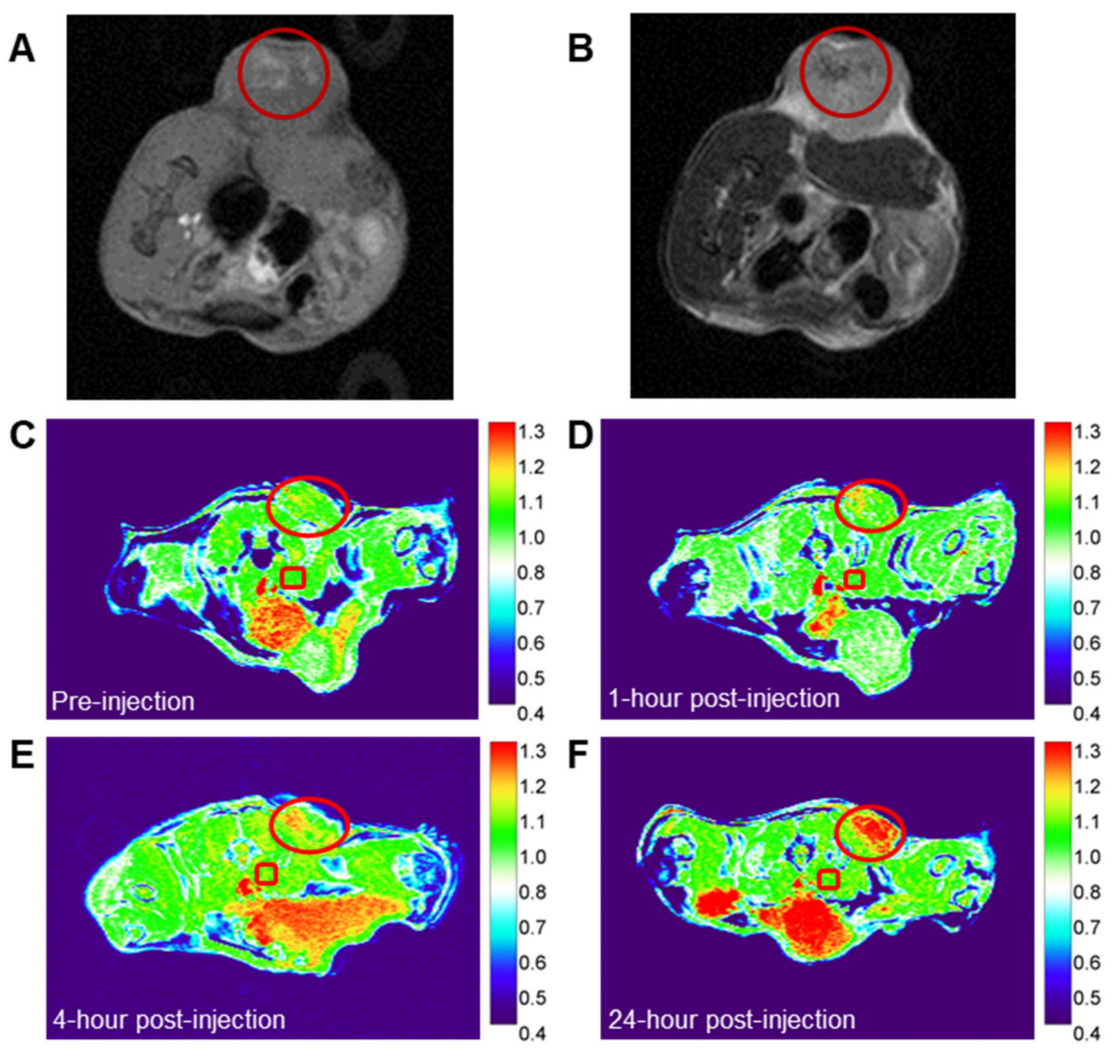
***In vivo* MR imaging of Ti-Gd-TA NPs.** (A) T_1_-weighted (B) T_2_-weighted images after intratumoral injection of the NP sol-gel solution into multiple regions of 4T1 tumor in C57BL/6 mouse. (C-F) T_1_-weighted images of 4T1 tumor-bearing C57BL/6 mouse at different time points after the intravenous injection of Gd-Ti-FA-TA NPs. Circle = tumor area; Square = reference tissue for normalizing intensity changes over time (see text).

The chelation of Gd with TA enhances both *r*_1_ and *r*_2_ relaxivities, enabling the potential use of Gd-Ti-FA-TA NPs as dual-modality T_1_ and T_2_ MRI contrast agents. To assess this capability, we developed a 4T1 tumor-bearing C57BL/6 mouse model and administered Gd-Ti-FA-TA NPs both intratumorally and intravenously. For intratumoral injection, we utilized a sol-gel material prepared with 20 wt% Pluronic F127, which transitions from liquid at room temperature to a gel at 37 °C 55, 56. Injection of 40 μL of the formulation from a solution of 100 μg/mL Gd-Ti-FA-TA NPs into multiple tumor regions allowed the NPs to persist in the tumor area for over 72 h. Using T_1_- and T_2_-weighted sequences on a 3 T scanner, we demonstrated that Gd-Ti-FA-TA NPs enhanced both T_1_ (bright)- and T_2_ (dark)-weighted contrast in the tumor (Fig. 6 A,B). To evaluate systemic administration, we injected via the mouse tail vein Gd-Ti-FA-TA NPs (100 μL of 1 mg/mL in PBS; n = 3) at different time points. We used the same tumor model and 3 T preclinical MRI scanner to perform imaging with a T_1_-weighted 2D gradient echo sequence (Fig. 6 C-F). Image intensity changes in the tumor (red circle) were normalized to the average intensity of the skeletal muscle near the spine in each image, which was not expected to change over time (red square). At the pre-injection point, the tumor intensity was similar to the reference but progressively intensified over time. The accumulation of Gd-Ti-FA-TA NPs in the tumor was visible within 1 h post-injection, attaining the highest contrast observed intensity at 24 h time point the mice were euthanized. These *in vivo* results confirm the potential of using the pro-theranostic Gd-Ti-FA-TA NPs for dual-modality MRI, providing an effective strategy for deep tissue imaging and MRI-guided RaST *in vivo*.

### Radionuclide stimulates pro-theranostic Gd-Ti-FA-TA NPs to inhibit tumor progression *in vivo*

**Figure 7:**
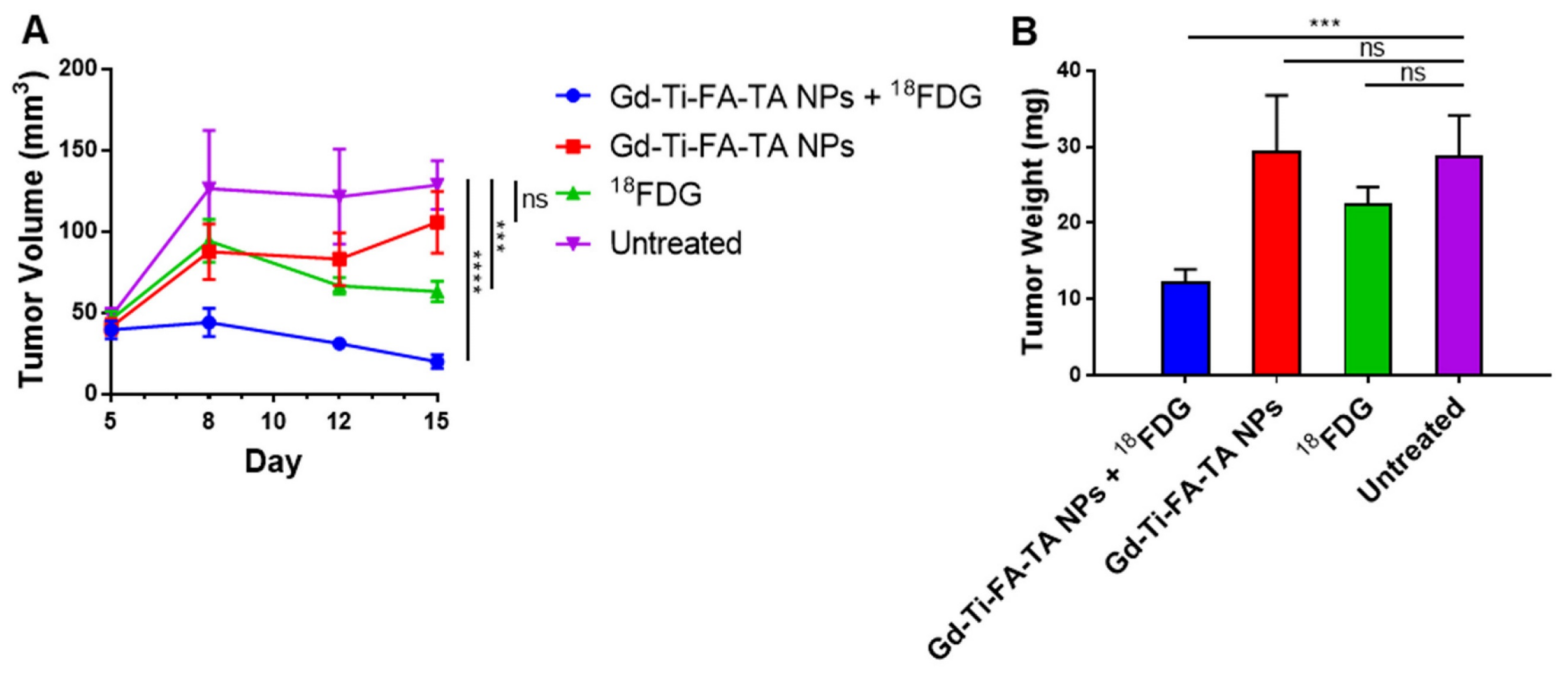
***In vivo* RaST study.** (A) Tumor volumes by means in different groups through day 5 to day 15, *p*(^18^FDG) = 0.0039, *p*(Gd-Ti-FA-TA NPs) < 0.0001. (B) Tumor weights in different groups after euthanasia on day 16, *p =* 0.0056.

A combination of enhanced ROS-generating capacity of Gd-Ti-FA-TA NPs, potentiation of cellular response to RaST, and uptake in tumors *in vivo* suggests that it could serve as a cancer-targeted RaST agent *in vivo*. Toward this goal, we treated C57BL/6 mice bearing subcutaneous 4T1 tumors with Gd-Ti-FA-TA NPs and ^18^FDG (RaST), Gd-Ti-FA-TA NPs alone, and ^18^FDG alone and compared the results against an untreated control group. Caliper measurements of tumor volume over time showed similar tumor progression patterns between mice treated with Gd-Ti-FA-TA NPs alone and the untreated control (Fig. 7A). Excised tumor weights at the study endpoint (Day 15) were similar for both the untreated and Gd-Ti-FA-TA NPs alone cohorts (Fig. 7B), supporting the longitudinal tumor volume measurement data. Mice treated with ^18^FDG alone initially exhibited a decrease in the tumor volume, followed by tumor growth stasis. However, the tumor weight at the study endpoint was not significantly different from the Gd-Ti-FA-TA NPs and untreated groups (Fig. 7B). Efforts to use ^18^FDG for cancer treatment (positherapy) at much higher activity doses than what was used in this study have been reported in rodents 57 and patients 58. Despite these reports, the poor efficacy of cancer positherapy has prevented further clinical trials. Moreover, a recent study demonstrated that some tumors initially respond to ^18^FDG positherapy, but they all relapsed, demonstrating a transient tumor inhibition effect 51. In contrast, mice administered with RaST components (Gd-Ti-FA-TA NPs + ^18^FDG) prevented tumor progression, decreasing to 15% of tumor volumes of the untreated group. The corresponding tumor weight showed that RaST significantly inhibited tumor progression compared to other treatment and untreated groups (*p =* 0.0056). These findings underscore the potential of using Gd-Ti-FA-TA NPs as a multimodal imaging RaST agent.

BLI was also used to monitor tumor growth, but the signal decreased over time in all cases, including the untreated control, and no significant difference between the groups was observed on day 15 (Fig. S8). The loss of BLI in the C57BL/6 mouse model suggests that these immune-competent mice stimulate a cellular response against luciferase 59. This can lead to the proliferation of cells devoid of the reporter, which can compromise data analysis. Further, 4T1 tumor cells, which originated from BALB/c mice, could induce an allogeneic response in C57BL/6 mice 60.

## Conclusion

We developed and characterized new TA-based monodispersed NPs for pro-theranostic applications. Integrating Gd and Ti into a TA framework, combined with folate receptor targeting and NIR fluorescence, offers a multifunctional platform for enhanced imaging and therapeutic applications. The chelation of Gd with TA significantly enhanced both *r*_1_ and *r*_2_ relaxivities, improving the MRI contrast capabilities of the NPs compared to commercial Gd-DTPA. We found that these materials exhibit magnetic field-dependent relaxivities, demonstrating the versatility of these NPs in various MRI settings, including both T_1_- and T_2_-weighted imaging modalities. The higher *r*_2_/*r*_1_ relaxivity ratio at increased magnetic fields indicates a strong potential for T_2_-weighted MRI, while still providing adequate T_1_ contrast enhancement at lower fields.

The inclusion of Ti in the NPs enhanced their ROS-generating capability, which was further potentiated by UV light and Cerenkov-radiating radionuclides. This dual functionality of ROS generation and imaging highlights the therapeutic potential of the NPs, particularly in strategies such as RaST. *In vitro* and *in vivo* experiments demonstrated the effective targeting and accumulation of Gd-Ti-FA-TA NPs in tumor cells and tissues, facilitated by folate receptor-mediated endocytosis. The intratumoral and intravenous administration routes showed significant tumor retention and enhanced imaging contrast, confirming MRI and fluorescence imaging in the same NPs for shallow and deep tissue imaging. The successful implementation of RaST *in vivo* using Gd-Ti-FA-TA NPs and ^18^FDG suppressed the tumor progression. Taken together, this study demonstrates the potential of Gd-Ti-FA-TA NPs as a versatile platform for enhancing cancer imaging and therapy, paving the way for future developments in multifunctional image-guided therapeutic interventions, where MRI will be used to determine NP distribution and timing of radionuclide administration to achieve localized, precision RaST.

## Supplementary Material

Supplementary figures.

## Figures and Tables

**Table 1:** Relaxivities of Gd-TA associated NPs and reference Gd-DTPA at different field strengths

Magnetic field strength	Gd-Ti-FA-TA			Gd-FA-TA			TiO2-Gd-FA-TA			Gd-DTPA		
	*r*_1_	*r*_2_	*r*_2_/r_1_	*r*_1_	*r*_2_	*r*_2_/*r*_1_	*r*_1_	*r*_2_	*r*_2_/*r*_1_	*r*_1_	*r*_2_	*r*_2_/*r*_1_
1.4 T	25.6	41.0	1.60	34.3	63.2	1.84	23.6	34.1	1.45	3.60	4.30	1.19
3 T	20.8	72.1	3.47	30.7	66.6	2.17	18.8	67.0	3.56	4.83	4.90	1.02
4.7 T	8.89	63.8	7.18	14.0	71.1	5.08	9.31	56.0	6.02	4.57	6.81	1.49
7 T	2.79	36.1	12.9	3.34	59.4	17.8	3.14	44.9	14.3	4.49	5.21	1.16

## Abbreviations

NP: nanoparticle
TA: tannic acid
TiO_2_: titanium dioxide
ROS: reactive oxygen species
FDG: 2-fluorodeoxyglucose
RaST: radionuclide-stimulated dynamic therapy
MRI: magnetic resonance imaging
NIR: near-infrared
FA: folic acid
PVP: poly(vinyl pyrrolidone)
ATP: adenosine triphosphate
MTT: 3-(4, 5-dimethylthiazolyl-2)-2, 5-diphenyltetrazolium bromide
Gd-DTPA: gadolinium-diethylenetriamine pentaacetic acid
TEM: transmission electron microscopy
DLS: dynamic light scattering
PDI: polydispersity index
UV-vis: ultraviolet-visible
XRD: X-ray diffraction
XPS: X-ray photoelectron spectroscopy
EPR: enhanced permeability and retention
IC_50_: half-maximal inhibitory concentration
IV: intravenous
BLI: bioluminescence imaging
