# Supplementary material for: Cancer-targeted pro-theranostic bi-metallic organo-coordination nanoparticles
Source: Theranostics. 2025 Jan 1;15(4):1205–20. doi: 10.7150/thno.99863 (PMC11729564; doi:10.7150/thno.99863)
Supplement: Supplementary file 1 — Supplementary figures. [file thnov15p1205s1.pdf]

Supplementary materials

Cancer-targeted pro-theranostic bi-metallic organo-coordination nanoparticles

Hengbo Huang,[1,2] Lei Fang,[1] Janaka Wansapura,[3] Julie L. Prior,[1] Brad Manion,[1] Baogang Xu,[1,4] Cody Hongsermeier,[4] Nisha Gamadia,[4] Nicole Blasi,[4] Rui Tang,[1] Christopher Egbulefu,[1,4] Monica Shokeen,[1] James D. Quirk,[1] Samuel Achilefu[1,2,4] *

[1]Departments of Radiology, Washington University in St. Louis, MO 63110, USA.

[2]Departments of Biomedical Engineering, Washington University in St. Louis, MO 63110, USA.

[3]Advanced Imaging Research Center, University of Texas Southwestern Medical Center, Dallas, TX 75390, USA

[4]Department of Biomedical Engineering, University of Texas Southwestern Medical Center, Dallas, TX 75235, USA

***Corresponding author:** Samuel Achilefu (samuel.achilefu@utsouthwestern.edu)

This file includes:

Figures S1 to S8

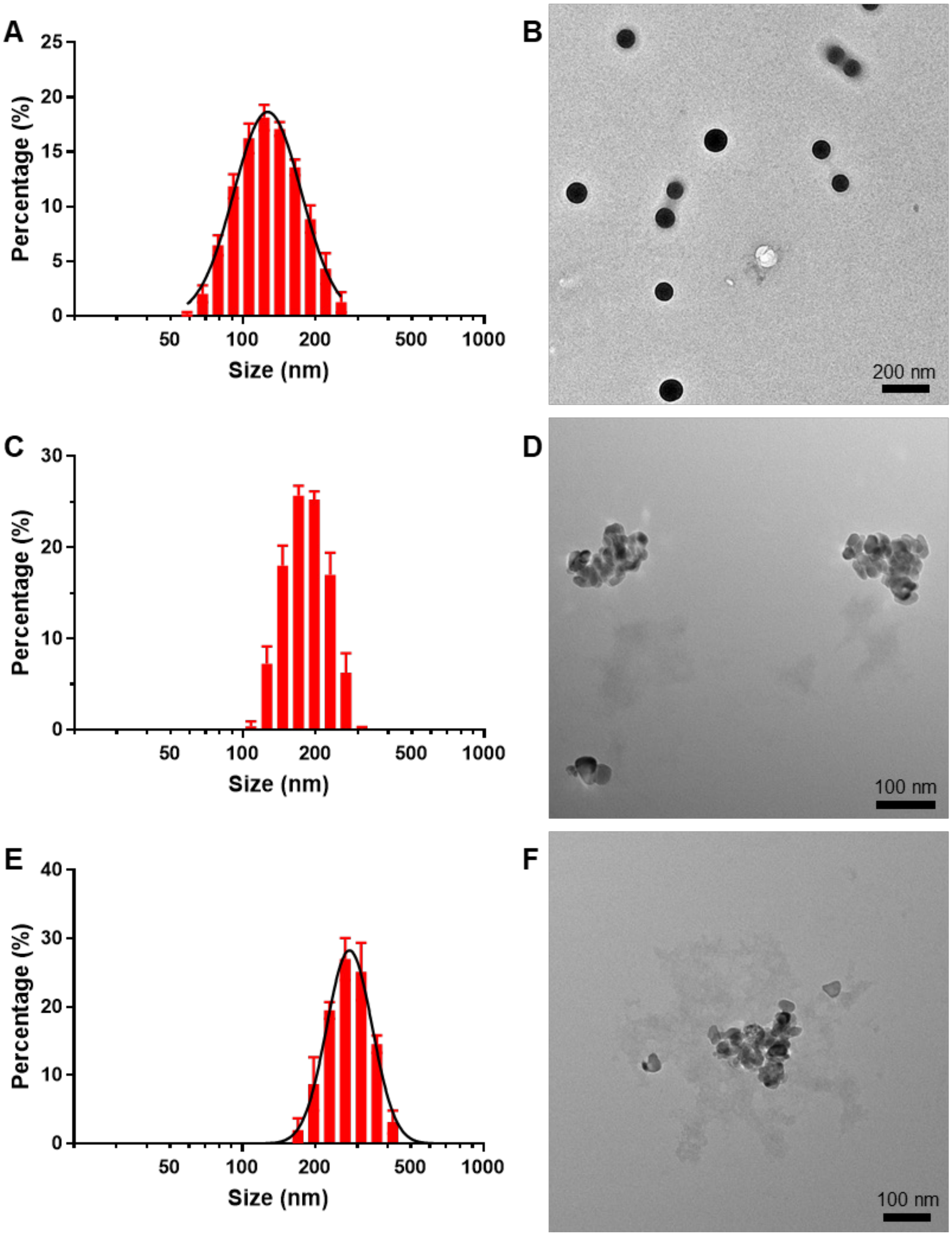

Figure S1. DLS and representative TEM images of Gd-FA-TA NPs, TiO_2 anatase, and TiO_2-Gd-FA-TA NPs. (A) Gd-FA-TA NPs particle size determination by DLS. (B) Representative TEM images of Gd-FA-TA NPs. (Scale bar: 200 nm.) (C) TiO_2 anatase particle size determination by DLS. (D) Representative TEM images of TiO_2 anatase. (Scale bar: 100 nm.) (E) TiO_2-Gd-FA-TA NPs particle size determination by DLS. (F) Representative TEM images of TiO_2-Gd-FA-TA NPs. (Scale bar: 100 nm).

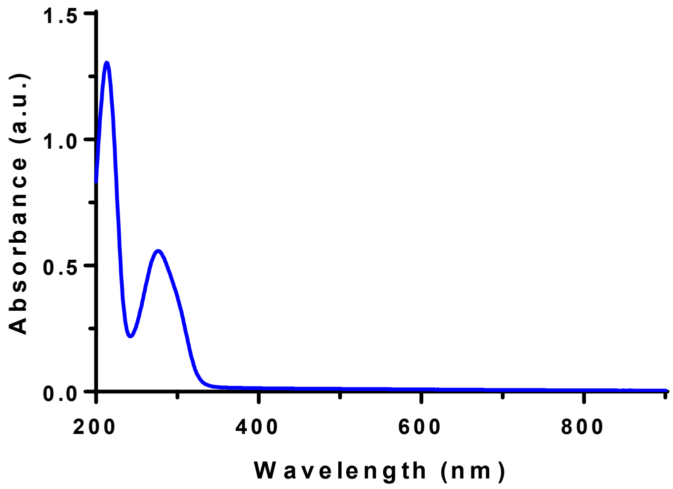

Figure S2. UV-vis absorption spectra of tannic acid (TA).

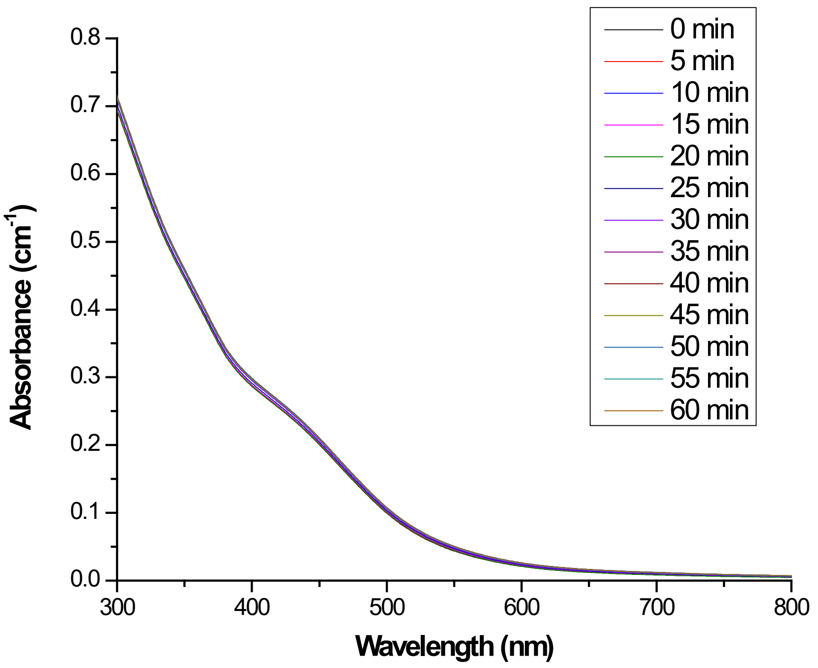

Figure S3. The absorption spectra of Gd-Ti-FA-TA NPs after UV light irradiation for 5-60 min.

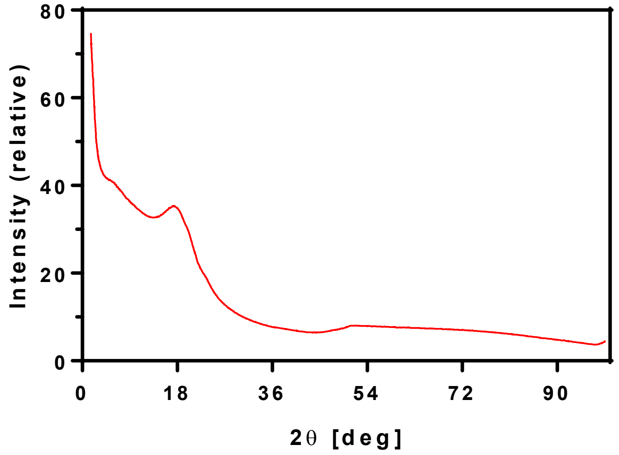

Figure S4. XRD spectrum of Gd-Ti-FA-TA NPs.

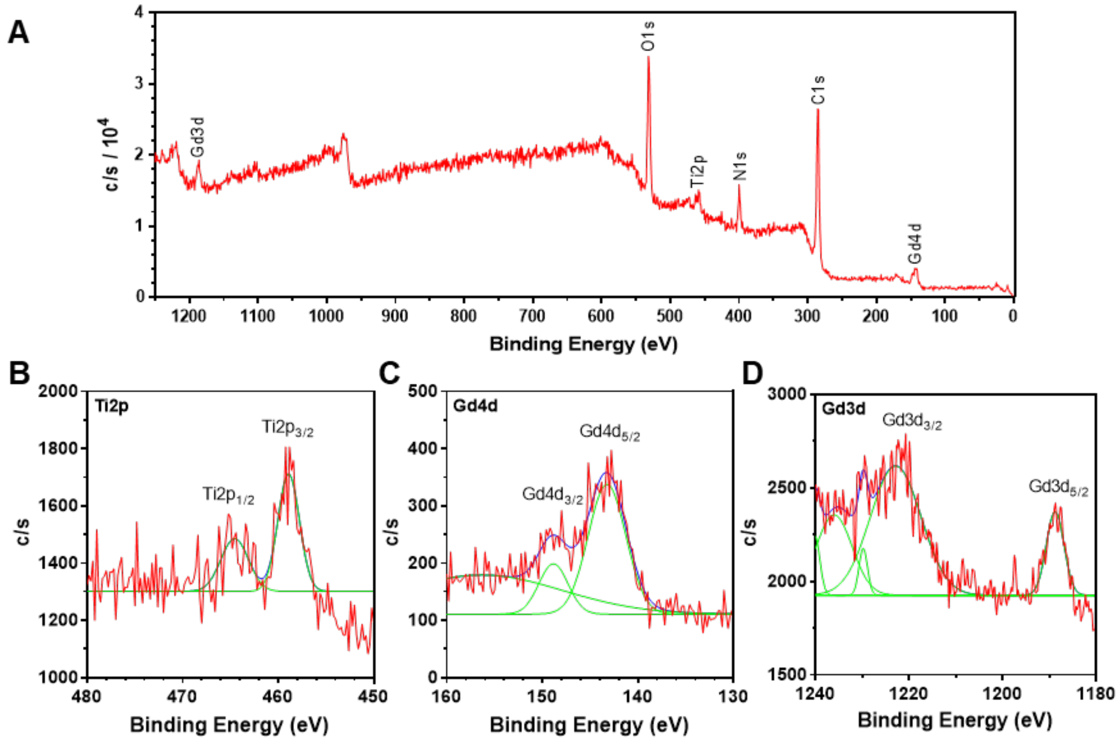

Figure S5. XPS spectra for Gd-Ti-FA-TA NPs, (A) Survey spectra, (B) Ti 2p, (C) Gd 4d, and (D) Gd 3d spectra.

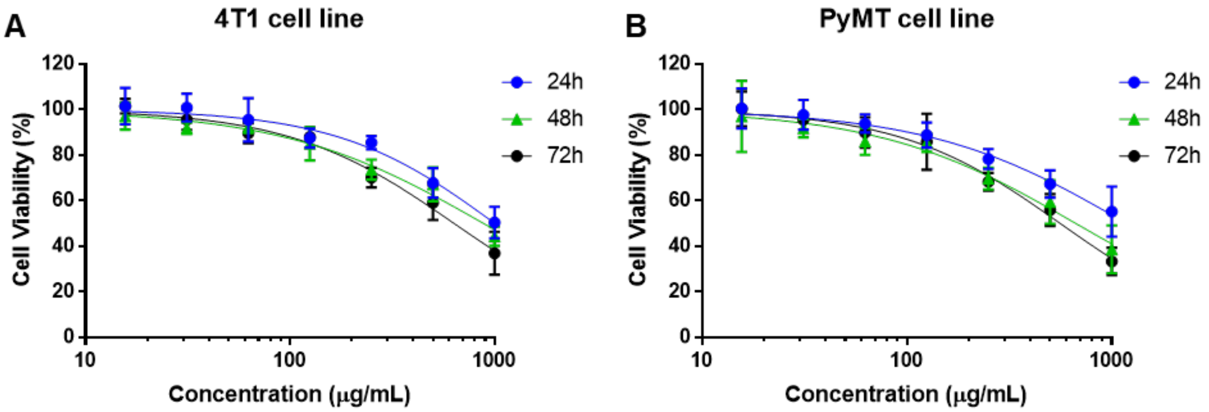

Figure S6. (A) 4T1 and (B) PyMT-Bo1 cell viability after incubating with Gd-Ti-FA-TA NPs using MTT assay. The concentration of Gd-Ti-FA-TA NPs ranged from 15.6 μg/mL to 1,000 μg/mL. The viability was measured at 24 h, 48 h, and 72 h time points.

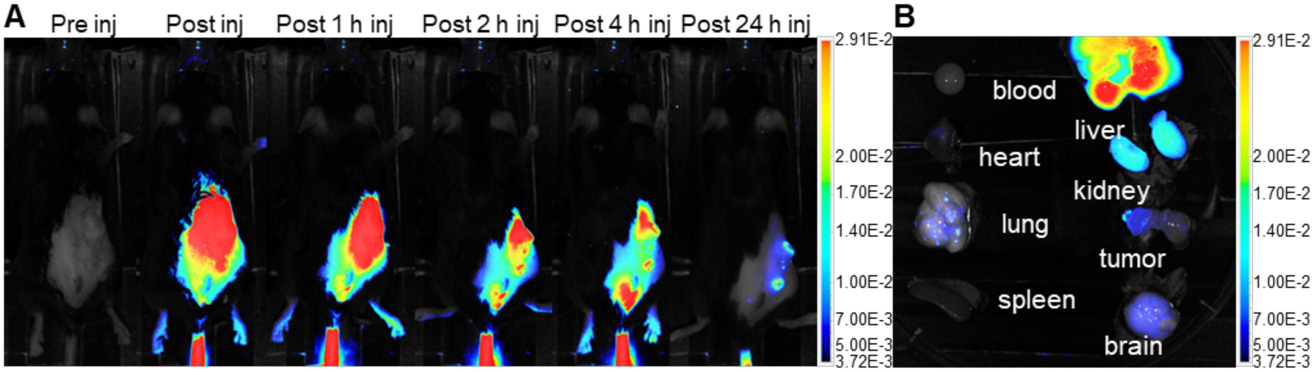

Figure S7. *In vivo* and *ex vivo* fluorescence imaging of cypate-loaded Gd-Ti-FA-TA NP distribution. (A) Dorsal view fluorescence images of representative mouse after tail vein injection of cypate-loaded Gd-Ti-FA-TA NPs. Images were acquired pre-injection, immediately post-injection, and 1, 2, 4, and 24 h post-injection. (B) Ex vivo fluorescence biodistribution of major organs after mouse euthanasia 24 h post-injection of the NPs.

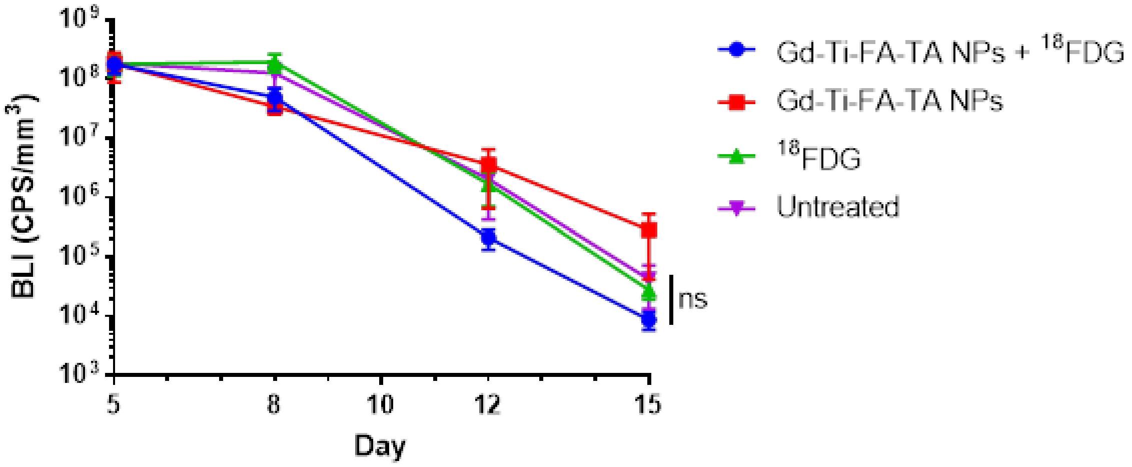

Figure S8. BLI intensity in untreated and different treatment groups from day 5 to day 15.
